# Non‐Invasive Brain Stimulation of the Ventromedial Prefrontal Cortex Improves Behavioral Inhibition by Enhancing the Processing Depth and Anticipation of Outcomes in a Gambling Task

**DOI:** 10.1111/psyp.70227

**Published:** 2026-01-09

**Authors:** Thomas Kroker, Maimu Alissa Rehbein, Miroslaw Wyczesany, Riccardo Bianco, Alejandro Espino‐Paya, Markus Junghöfer

**Affiliations:** ^1^ Institute for Biomagnetism and Biosignalanalysis, University of Muenster Muenster Germany; ^2^ Otto Creutzfeldt Center for Cognitive and Behavioral Neuroscience, University of Muenster Muenster Germany; ^3^ Institute of Psychology, Unit of Clinical Psychology and Psychotherapy for Children and Adolescents University of Osnabrueck Osnabrueck Germany; ^4^ Institute of Psychology, Jagiellonian University Krakow Poland

**Keywords:** addiction, behavioral inhibition, EEG, gambling, transcranial direct current stimulation, ventromedial prefrontal cortex

## Abstract

Disordered gambling and other behavioral addictions are characterized by a lack of behavioral inhibition, such that patients repeatedly make disadvantageous decisions and fail to disengage from maladaptive behavior. Previous research has shown that behavioral addictions are associated with altered ventromedial prefrontal cortex (vmPFC) activity, making it an interesting target for neuromodulation. Using a gambling paradigm containing positive and negative expected value trials, we investigated decision‐making and feedback processing while stimulating the vmPFC via transcranial direct current stimulation, and we recorded neural responses via EEG. We recorded behavioral and neural responses when the cue indicating reward probability and the outcome (gain/loss) were presented. At the behavioral level, interactions of stimulation by cue modulated gambling behavior, whereby we found different patterns for positive and negative expected value trials. We observed the respective interactions in the EEG data covering left dlPFC and parietal areas. The stimulation modulated the processing of outcomes depending on its probability in the behavioral and neural data. The behavioral results suggest improved gambling behavior after vmPFC excitation, especially when the risk of losing is high, visible in enhanced behavioral inhibition. This appears to be due to an enhanced anticipation based on reward probability and processing depth of outcomes. The neural results indicate that vmPFC excitation allows for a better ability to suppress high‐risk decisions and a more accurate updating of gambling‐related information. This makes excitatory vmPFC‐tDCS promising as an additional treatment option for behavioral addictions.

## Impact Statement

This study demonstrates that excitatory transcranial direct current stimulation (tDCS) of the ventromedial prefrontal cortex (vmPFC) can improve decision‐making in a gambling context by enhancing behavioral inhibition and anticipation of outcomes. Importantly, learning from gains works differently than learning from losses. Neural and behavioral data suggest vmPFC stimulation reduces risky choices and supports better updating of reward‐related information. These findings highlight vmPFC‐tDCS as a promising neuromodulatory intervention for behavioral addictions.

## 1 Introduction

Impulse control disorders have received increasing scientific attention in recent years, partly because more stimuli exist in our environment—such as social media, online gambling, and pornography—that trigger such behavior, resulting in an increased prevalence of these disorders (Meng et al. 2022). In fact, the intensified study of such disorders has resulted in a regrouping in the ICD‐11, such that many disorders that were classified as impulse control disorders in the ICD‐10 have now been recoded as behavioral addictions (e.g., Gambling disorder, social media use disorder) (Brand et al. 2022, 2024; Grant et al. 2014). Thus, we will use the term *behavioral inhibition* instead of *impulse control* when referring to the behavior around such disorders.

To study the neural processes that underlie behavioral inhibition, gambling paradigms have frequently been used in healthy participants (DeMartino et al. 2006; Vicent et al. 2019) and in patients (van Holst et al. 2010a, 2010b; Zack et al. 2016). Neurobiological models of (pathological) gambling identify subcortical regions such as the ventral striatum, ventral tegmental area, and the amygdala as important players in reward processing and approach behavior, and they also include prefrontal areas that exert top‐down control and ensure proper behavioral inhibition, e.g., when an incautious impulse must be inhibited (Antons et al. 2020; Brand 2022). In this context, the ventromedial prefrontal cortex (vmPFC) and the dorsolateral prefrontal cortex (dlPFC) are particularly relevant. Both of these areas seem to be involved in behavioral inhibition, as observed in numerous fMRI studies (Boes et al. 2009; Chen et al. 2018; Sebastian et al. 2014; Steinbeis et al. 2012; Williams and Potenza, n.d.), and lesions in these regions result in an impaired ability to disengage from maladaptive behavior (Bechara, Damasio, and Damasio 2000; Bechara, Tranel, and Damasio 2000; Damasio 1996; Kroes et al. 2019). Furthermore, their strong functional and anatomical interconnectivity seems to play an important role as stimulation of these regions results in similar effects (Dantas et al. 2023). However, the underlying mechanisms behind their role in behavioral inhibition are not yet clear.

Therefore, this work tries to elucidate these mechanisms by combining two neuroscientific techniques to derive clinically useful implications. Specifically, by using electroencephalography (EEG), which has a high temporal resolution, in conjunction with an underlying source model, we aim to explain the reciprocal processes between the vmPFC and dlPFC, which are known to be anticorrelated (task‐positive network and default mode network) (Cheng et al. 2020; Smallwood et al. 2021). In this regard, previous EEG work has shown that the neural interplay between the vmPFC and dorsal prefrontal regions is important for anticipating outcomes and adapting consequences, i.e., maintaining one's current strategy or switching to a new one (Adelhöfer and Beste 2020; Domenech et al. 2020). As such, prediction errors appear to play a particularly important role in learning from previous outcomes (Domenech et al. 2020). Additionally, EEG correlates of vmPFC activity seem to be important for inhibiting maladaptive behaviors, such as substance abuse (Knyazev 2007).

Stimulating certain areas of the brain using transcranial direct current stimulation (tDCS) allows researchers to make causal inferences about neural functions, which is not possible in pure neuroimaging studies (Nitsche et al. 2008; Polanía et al. 2018). Furthermore, tDCS is also a therapeutic option for mental disorders (Hausman et al. 2024; Manuel et al. 2019; Marković et al. 2021; Vicario et al. 2020; Vöckel et al. 2024), and using tDCS to excite the vmPFC has been shown to improve behavioral inhibition in healthy (Ouellet et al. 2015; Boggio et al. 2010) and clinical populations (Boggio et al. 2010; Lapenta et al. 2018; Sergiou et al. 2022; Vöckel et al. 2024). In line with these findings, reducing the excitability of the vmPFC via tDCS or transcranial magnetic stimulation (TMS) has been found to result in impaired behavioral inhibition (Juan and Muggleton 2012). As patients with behavioral addictions show reduced activity in the vmPFC (Grant et al. 2010; van Holst et al. 2010a), a promising strategy in treating such addictions might be to excite this region with non‐invasive brain stimulation techniques. Importantly, this does not mean that the vmPFC is not involved in reward processing, as the vmPFC gets active when receiving a reward. However, the vmPFC does not become active before a high‐risk decision is made, whereas the nucleus accumbens does (Kuhnen and Knutson 2005). Regarding the dlPFC, there are also numerous papers using non‐invasive brain stimulation showing its involvement in behavioral inhibition (e.g., Oldrati et al. 2016). Interestingly, differences were found in dlPFC functioning between the hemispheres, i.e., right dlPFC excitation increased reflective/non‐impulsive thinking, whereas left dlPFC excitation did not (Edgcumbe et al. 2019). In addition, the effects of dlPFC were cumulative to a certain degree over multiple sessions of dlPFC‐tDCS (Edgcumbe et al. 2024).

In previous research, we showed that vmPFC excitation can improve decision‐making and strengthen behavioral inhibition via improved affective learning (Kroker et al. 2025), which was reflected in participants' reduced cognitive biases in a gambling task (Kroker et al. 2022; Kroker, Rehbein, et al. 2023; Kroker, Wyczesany, et al. 2023). However, our paradigms thus far have lacked the ecological validity to apply vmPFC‐tDCS to disordered gambling (or other behavioral addictions) in a clinical setting because, in our previous studies, participants could not lose money (they could only fall back to zero), i.e., our study designs had positive expected values only. However, actual gambling typically has a negative expected value, as one would almost always expect to lose money when playing for a relatively long period of time. Thus, we developed a paradigm containing positive and negative expected trials to investigate whether learning and anticipation of consequences differs between pleasant stimuli (gains) and unpleasant stimuli (losses). Notably, our preliminary work on unpleasant stimuli has shown that vmPFC stimulation improves learning from fear‐related stimuli (Kroker et al. 2025; Roesmann et al. 2021).

**FIGURE 1:**
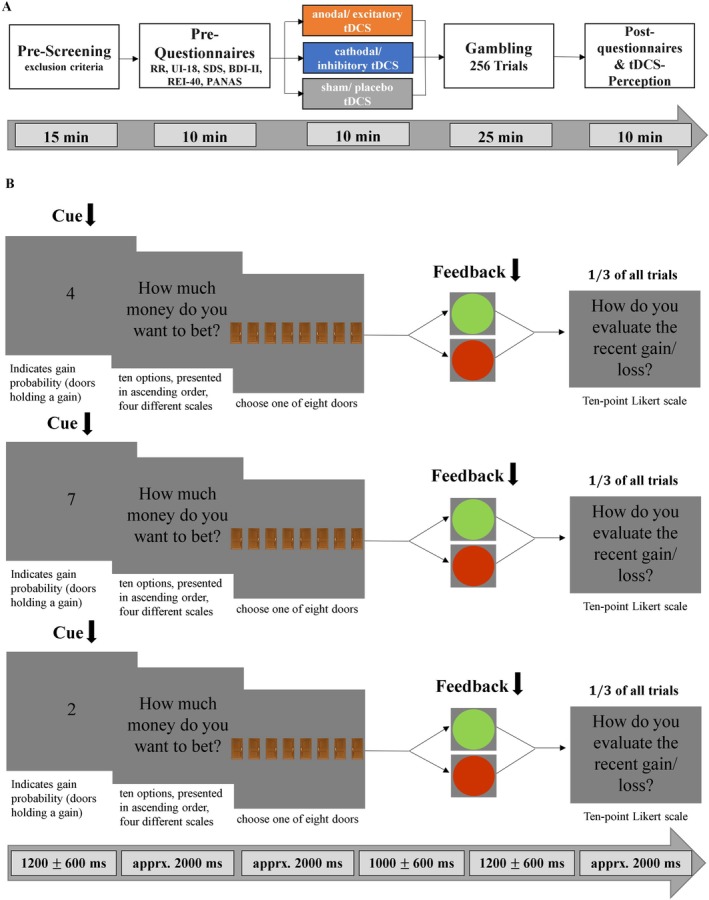
(A) Overview of experimental procedure of the reward/gambling paradigm. In a between‐subject design, 72 healthy participants were pseudorandomly assigned to three stimulation groups (anodal, cathodal, sham) and were matched regarding demographic and psychometric characteristics. After 10 min of the respective tDCS stimulation, participants performed a gambling task of 256 trials while event‐related potentials were measured by EEG. (B) Course of a single trial in the gambling task. Each trial began with a cue indicating the reward probability, i.e., how many of eight doors shown below would hold a gain. Next, participants were asked how much money they wanted to bet in the upcoming trial. After choosing one of the eight doors, participants received feedback on whether they won or lost the amount they had bet. Finally, in one‐third of the trials, participants were asked to rate the recent outcome. Arrows indicate EEG triggers.

Based on previous research, we hypothesize that in the ‘cue phase’, we will observe improved gambling behavior—i.e. participants will bet more money when the chance to win is high and bet less money when the risk of losing is high—after excitatory vmPFC‐tDCS. In this ‘cue phase’ participants need to weigh the chances and risks to decide how much money should be bet (see Figure 1B left). We expect this improvement to become evident in the trials with positive and negative expected values and develop over time. In the neural data (estimated based on event‐related potentials measured by electroencephalography), we expect that after excitatory vmPFC‐tDCS, we will find spatio‐temporal clusters that are associated with enhanced behavioral inhibition (interaction: stimulation × cue). The neural interaction pattern should indicate an enhanced inhibition of high‐risk choices after vmPFC excitation. In the feedback phase (Figure 1B right), we hypothesize that after excitatory vmPFC‐tDCS, we will observe an enhanced anticipation and processing depth of outcomes indicated by the feedback ratings. This will be indicated in a stronger consideration of the reward likelihoods indicated by the cues after excitatory stimulation in the feedback ratings. In the neural data (interaction: stimulation × reward probability × actual outcome), this should be reflected in stronger neural activity in response to unlikely outcomes, suggesting a greater need for reprocessing and potentially readjustment. In this paper we focus on the main and interaction effects involving stimulation; other effects are described in the Supporting Information.

## 2 Methods

### 2.1 Participants

We included 72 healthy participants between 19 and 28 years old (M = 23.24, SD = 3.44) who met our inclusion criteria (Section 1.1 in Supporting Information). Participants were pseudorandomly assigned to stimulation groups and matched regarding demographic criteria (see Table 1 top). Since interindividual differences in risk tolerance and decision‐making have a greater limiting effect in the design chosen here between subjects than in designs within subjects, we gathered relevant measures for intolerance of uncertainty (UI‐18, Gerlach et al. 2008), responsiveness of reward (den Van Berg et al. 2010), social desirability (SDS‐CM; Social Desirability Scale, Crowne and Marlowe 1960), and preferences for information processing (Rational‐Experiential Inventory‐40; Pacini and Epstein 1999). Furthermore, we gathered the Positive and Negative Affective Schedule (PANAS; Watson et al. 1988) to detect effects of current mood on decision‐making. The experimental groups did not differ regarding the above psychometric characteristics. They did not differ in their individual perception of the tDCS stimulation (see Table 1).

**TABLE 1:** Demographic and psychometric characteristics as well as rated tDCS perception of participants in the anodal/excitatory, cathodal/inhibitory, and sham/placebo condition.

Variable	Excitatory		Inhibitory		Sham		Test	df_n	df_d	*p*
	M/N	SD	M/N	SD	M/N	SD	*F*/*χ* ^2^			
Demographic characteristics										
*N*	24	—	24	—	24	—	*χ* ^2^ = 0.00	2	—	1
Age	23.13	4.41	23.25	2.99	23.63	2.89	*F* = 0.13	2	68	0.879
Sex (female)	13	—	13	—	12	—	*χ* ^2^ = 0.05	2	—	0.974
Psychometric characteristics										
UI‐18	45.34	14.58	42.71	12.10	41.87	10.98	*F* = 1.28	2	68	0.286
RR	24.47	3.14	23.75	3.58	24.75	2.67	*F* = 0.64	2	68	0.529
SDS	12.02	3.41	13.22	3.06	13.05	3.54	*F* = 0.75	2	68	0.524
BDI‐II	3.52	3.19	3.25	2.24	2.58	2.03	*F* = 0.23	2	68	0.799
REI‐40	119.21	11.28	120.24	12.53	117.31	10.86	*F* = 0.08	2	68	0.775
PANAS										
Positive	30.04	5.49	29.91	6.46	30.20	6.88	*F* = 0.13	2	68	0.987
Negative	12.54	2.78	13.04	3.75	13.67	3.30	*F* = 1.24	2	68	0.659
tDCS perception										
Pleasantness	4.43	1.57	4.35	1.29	4.59	1.57	*F* = 1.56	2	68	0.236
Intensity	1.71	0.77	1.85	0.91	1.52	1.01	*F* = 1.78	2	68	0.184

*Note:* The post‐stimulation questionnaire assessed participants' perception of the tDCS stimulation. It asked, “How did you perceive the stimulation?” where a response was given on a six‐point Likert scale ranging from 1 (“very uncomfortable”) to 6 (“very comfortable”); it also asked, “How intense was your perception of the stimulation?” where a response was given on a six‐point Likert scale ranging from 1 (“very mild”) to 6 (“very intense”). Divergent degrees of freedom are due to missing values.

Abbreviations: BDI‐II, Beck Depression Inventory‐II (Beck et al. 1996); PANAS, Positive and Negative Affect Schedule (Watson et al. 1988); REI‐40, Rational‐Experiential Inventory‐40 (Pacini and Epstein 1999); RR, Reward responsiveness (den Van Berg et al. 2010); SDS, Social Desirability Scale (Crowne and Marlowe 1960); UI‐18, Intolerance of Uncertainty Scale‐18 (Gerlach et al. 2008).

The study was approved by the ethics committee of the medical school of the University of Muenster.

### 2.2 Procedure and Experimental Tasks

Participants received either excitatory, inhibitory, or sham stimulation of the vmPFC before they performed a monetary gambling task while being recorded by an EEG (Figure 1A). After filling in the questionnaires, participants received the stimulation. Next, the EEG cap was fitted to the participant's head and the gambling started, whereby the time between stimulation and gambling was minimized.

Each trial started with presenting the cue, which showed the chance of winning/risk of losing, i.e., a number between one and seven, indicating how many of the following eight doors contained a “gain.” This resulted in gain probabilities of 18, 28 … 68 and 78, so that some trials had a positive expected value (cues 5–7) and others a negative expected value (cues 1–3). Cue 4 resulted in an expected value of zero, as the probability to win or lose was 50%. After knowing the reward probabilities, participants were asked to choose how much money they wanted to bet. To make the task less transparent, in each trial participants could chose bets from four different scales each with 10 intermediate options with low (2, 4, 6, …, 20 cents), intermediate low (5, 10, 15, …, 50 cents), intermediate high (10–100 cents) and high (20, 40, 60, …, 200 cents) gain or risk options, respectively. This increased complexity was meant to make the task more challenging, prevent repeated uniformly bets across trials, thereby increasing variance and thus improving the chances for the detection of stimulation effects. To maximize their wins, participants should bet more money when the expected value is positive (i.e., cues 5–7) and bet less money when the expected value is negative (i.e., cues 1–3). After selecting a door, participants were given feedback on whether they had won or lost the amount they had previously bet. Green and red circles indicated gains and losses, respectively, with the bet amount in the center. After every third trial, participants were asked to rate their recent outcome: “How do you evaluate the recent gain/loss?” on a 10‐point Likert scale between (1: “very negative” to 10: “very positive”). Event related potentials were related to the presentation of the cue (cue‐phase) and to the presentation of the feedback (feedback‐phase).

### 2.3 tDCS

**FIGURE 2:**
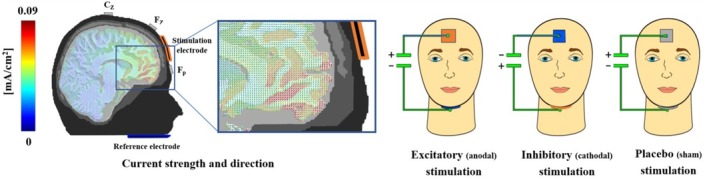
The use of an extra‐cerebral chin reference enabled virtually reference‐free stimulation of the vmPFC, facilitating clear differentiation between excitatory, inhibitory, and sham effects. Participants were stimulated for 10 min with 1.5 mA in the excitatory and inhibitory (anodal/cathodal forehead electrode) conditions, and for the sham condition participants were stimulated with 1.5 mA for 15 s each at the start and the end of a 10‐min period (the 9.5 min in between had zero current). The results of the simulation demonstrated that a 1.5‐mA stimulation would result in the highest current density in the vmPFC regions, reaching approximately 0.09 mA/cm^2^ (illustrated in red). In the actual application, the same colors were used for the sponges and cables to prevent participants from being able to use visual cues to infer their stimulation condition. This figure was initially published by Junghofer et al. (2017) in *Cerebral Cortex*.

We used a between‐subjects design with excitatory/anodal, inhibitory/cathodal, or sham/placebo stimulation. We applied the same tDCS setup as in our previous studies (Junghofer et al. 2017; Kroker et al. 2022; Kroker, Rehbein, et al. 2023; Kroker, Wyczesany, et al. 2023; Rehbein et al. 2023; Roesmann et al. 2021; Winker et al. 2018, 2019, 2020). The active electrode was placed on the forehead, while the reference electrode was placed under the chin, allowing us to eliminate a reference electrode over the brain as a possible confounding variable. The optimal forehead electrode position for maximal vmPFC stimulation and minimal adjacent stimulation was evaluated using finite element modeling (Wagner et al. 2014), which indicates a maximal stimulation intensity of the vmPFC and minimal stimulation of neighboring regions. Our computational modeling revealed that while stimulation focality at the vmPFC significantly increased with decreasing forehead electrode size, this effect was negligible for the quite remote chin reference. Thus, for focality we applied a smaller 3 × 3 cm electrode between the 10–20 electrode positions Fz and Fpz at the forehead and, to reduce current density especially in oral regions of no interest, a bigger 5 × 5 cm pad has been chosen as chin reference. We injected a current of 1.5 mA for 10 min in the active conditions but for only 30 s in the sham condition (Figure 2). The stimulation was delivered in a double‐blinded manner.

### 2.4 Recording and Preprocessing of EEG Data

The EEG data for the positive expected value and negative expected value paradigms were measured with a 128 whole‐head EEG sensor system (EGI cap, Electrical Geodesics, Oregon, USA) and preprocessed using the MATLAB‐based EMEGS software (Version 2.3; Peyk et al. 2011). Individual trials were processed and artifacts were corrected according to the method for statistical control of artifacts in high‐density EEG data (Junghöfer et al. 2000). If noisy channels were detected, their signals were estimated using spherical‐spline interpolation based on the weighted signals of all the other sensors. A minimum threshold of 0.01 was used to estimate the quality of the interpolation. Trials that exceeded this threshold were rejected. Participants were excluded from further analysis if more than 30% of the trials in the EEG baseline or test phase were rejected. Nine participants were excluded due to global cross‐conditional EEG artifacts (excitatory: *n* = 3; inhibitory: *n* = 4; sham: *n* = 2). After averaging, we estimated the underlying neural sources of the recorded event‐related potentials using L2 minimum‐norm‐estimates (Hämäläinen and Ilmoniemi 1994). The source model used a spherical head model with 350 evenly distributed dipole pairs positioned at 87% of the individually fitted head radius. Source topographies were computed using a Tikhonov regularization parameter of k = 0.1, yielding source‐direction‐independent neural activity for each participant, condition, and time point. For more details, please see Section 1.2 in Supporting Information.

### 2.5 Statistical Analysis

To test our hypotheses in the behavioral data, we used a mixed‐effects model and modeled random effects for stimulation. In the cue phase, we used stimulation (excitatory, inhibitory, sham), cue (1–7), and trial number (1–256) as predictors to examine the amount bet. Additionally, the actual outcome was analyzed with the predictors stimulation (excitatory, inhibitory, sham) and trial number (1–256). Since we had a special interest in the differences between the trials with positive and negative expected values, we examined effects involving stimulation in these two conditions separately. We performed a post hoc power analysis, considering the interaction effect of stimulation × expected value × trial number, with an effect size of partial *η* ^2^ = 0.14. This analysis revealed an achieved power of 0.92, based on the 72 participants recruited. In the feedback phase, we employed stimulation (excitatory, inhibitory, sham), reward probability (high chance/low risk (cues 5–7), low chance/high risk (cues 1–3)), and actual outcome (gain, loss) as predictors to analyze the feedback ratings after every third trial. The cues were combined here to harmonize the behavioral and the neural analyses; if we had kept all factor steps, we would not have received a sufficient signal‐to‐noise ratio for each condition. Again, we calculated a post hoc power analysis using the interaction stimulation × probability of actual outcome × actual outcome with an effect size of partial *η* ^2^ = 0.08. This suggested an actual achieved power of 0.69. The power analysis was conducted using G*Power (Faul et al. 2007).

The neural data was analyzed using ANOVAs, and a permutation‐based approach was applied to extract the clusters (Maris and Oostenveld 2007). The method first calculates statistical values at each neural source and time point, identifying points exceeding a sensor‐level threshold of *p* = 0.01 and grouping adjacent ones into spatio‐temporal clusters. Each cluster's magnitude, or cluster‐mass, is computed based on its statistical value, spatial extent, and temporal extent. Observed cluster‐masses are then compared against a null distribution from 1000 permutations, and clusters exceeding the 95th percentile (*p* = 0.05) are considered significant. For details, please consult the Supporting Information. In the cue phase, the factors stimulation (excitatory, inhibitory, sham) and cue (1–7) were used to investigate the neural responses. To analyze the neural data in the feedback phase, the factors stimulation (excitatory, inhibitory, sham), reward probability (high chance/low risk (cues 5–7), low chance/high risk (cues 1–3)), and actual outcome (gain, loss) were used.

## 3 Results

### 3.1 Cue Phase

#### 3.1.1 Behavioral Effects

**FIGURE 3:**
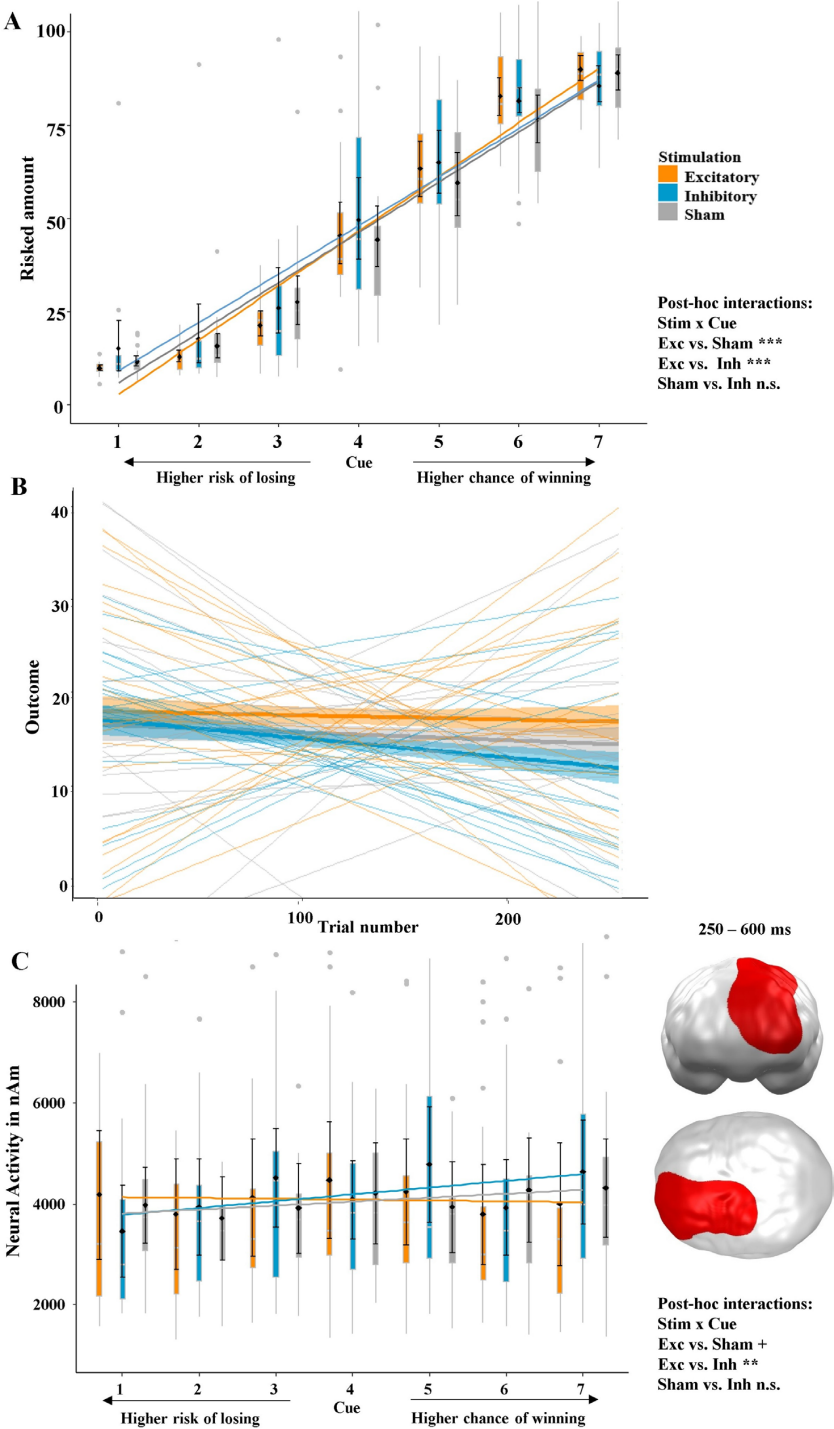
(A) Amount bet depending on stimulation condition and cue indicating reward probability. Gambling was more adaptive in the excitatory group, as more money was bet when the reward probability was high (positive expected value) and less money was bet when the risk of losing was high (negative expected value) compared to the sham and inhibitory stimulation groups. (B) Mean actual outcome per trial as a function of stimulation and trial number. Averaged regressions within stimulation groups are shown with bold lines, individual regressions with thin lines. (C) Significant spatiotemporal cluster in the left dlPFC featuring an interaction effect of stimulation × cue. Excitatory stimulation resulted in greater neural activity in response to high‐risk options, while the sham and inhibitory groups showed the opposite pattern. Boxplots indicate means (black dots), medians (gray lines) and lower and upper quartiles. Asterisks indicate significance levels: + < 0.1, ** < 0.01, *** < 0.001.

At the behavioral level, the mixed‐effects model using the predictors stimulation, cue, and trial number to analyze the amount bet revealed a significant overall model, when compared to the null model (*χ* ^2^(12) = 22,710, *p* < 0.001). The interaction of interest, namely stimulation × cue (*t* = 9.61, *p* < 0.001, partial *η* ^2^ = 0.06; Figure 3A), was also significant. Post hoc tests on the stimulation × cue interaction showed that the effect of cue differed significantly between excitatory and sham (*t* = 8.54, *p* < 0.001, partial *η* ^2^ = 0.02) as well as between excitatory and inhibitory conditions (*t* = 9.48, *p* < 0.001, partial *η* ^2^ = 0.03), but this effect was not significant between inhibitory and sham stimulation (*t* = 0.87, *p* = 0.386). Furthermore, the main effect of stimulation was significant, indicating more risk‐taking behavior after inhibitory vmPFC‐tDCS compared to sham and excitatory stimulation (*t* = −2.93, *p* = 0.005, partial *η* ^2^ = 0.15; Section 2.1 in Supporting Information). To replicate our previous results on improved learning after excitatory vmPFC‐tDCS (Kroker et al. 2025), we tested the interaction of stimulation × cue × trial number, which was insignificant (*t* = −1.06, *p* = 0.286). Interestingly, this was due to the opposite learning patterns that occurred in trials with positive and negative expected values, such that they canceled out each other (Figure 5). Although the interaction effect of stimulation × cue was significant, the main effect of stimulation on the overall outcomes did not reach significance (*t* = 1.06, *p* = 0.295).

#### 3.1.2 Neural Effects

At the neural level, a cluster in the left dlPFC and parietal regions was apparent, showing a significant interaction of stimulation × cue (*p* = 0.039). The post hoc mixed‐effects model revealed a highly significant interaction: *t* = 3.52, *p* < 0.001, partial *η* ^2^ = 0.08 (Figure 3C). Here, excitatory and sham conditions (*t* = 1.73, *p* = 0.084, partial *η* ^2^ = 0.01) and excitatory and inhibitory conditions (*t* = 2.68, *p* = 0.008, partial *η* ^2^ = 0.03) differed (trend‐) significantly, while the effect between inhibitory and sham conditions was insignificant (*t* = −1.02, *p* = 0.306). The neural activity in this cluster was positively correlated with the amount bet (*r* = 0.11, *p* = 0.024). Remarkably, this correlation was present in the inhibitory (*r* = 0.15, *p* = 0.039) and sham groups (*r* = 0.14, *p* = 0.040), but it was insignificant in the excitatory group (*r* = 0.05, *p* = 0.43).

#### 3.1.3 Positive Versus Negative Expected Value Trials

**FIGURE 4:**
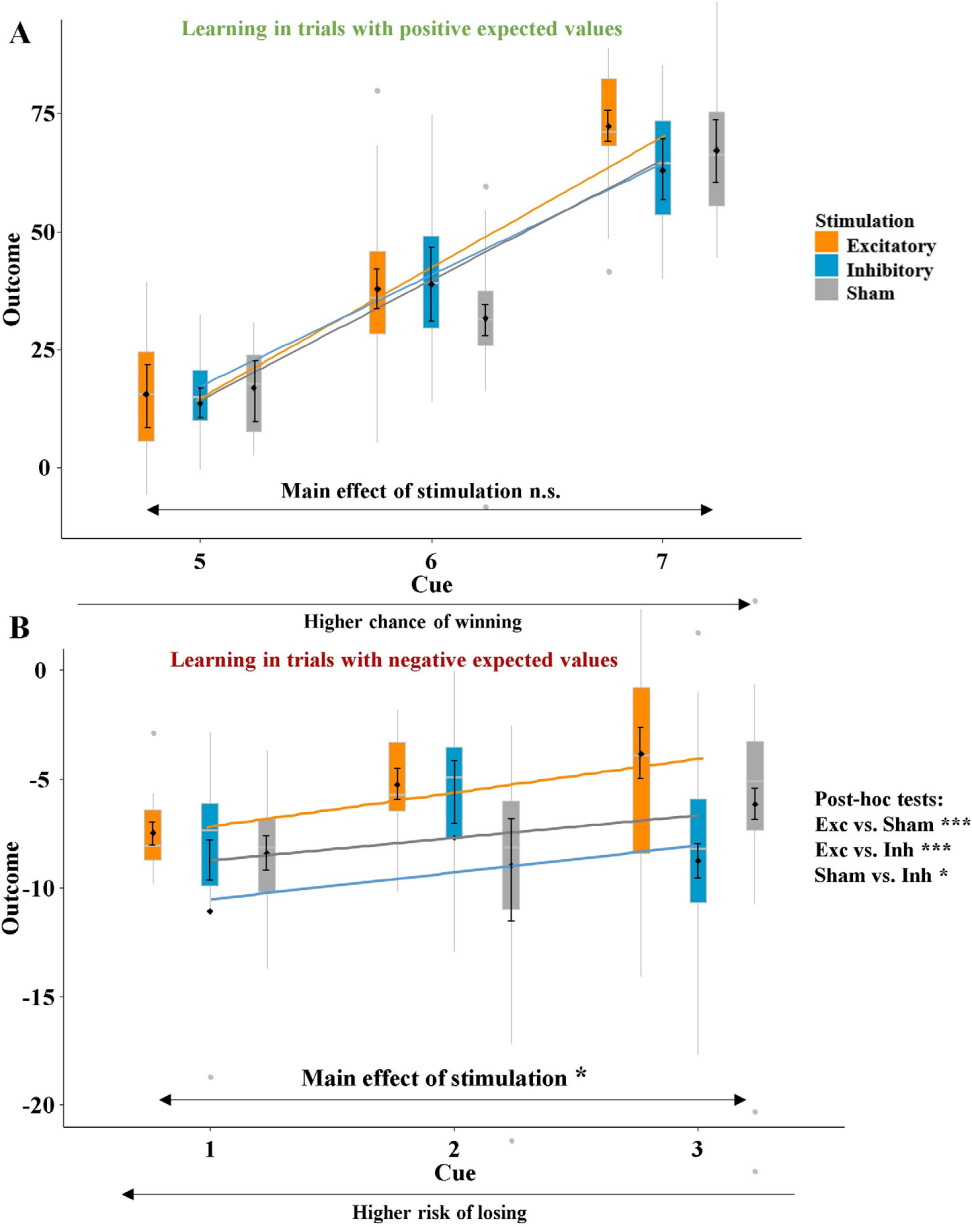
(A) Actual outcomes of trials with positive expected values. In this subgroup of trials, no difference between the stimulation conditions was present. (B) Actual outcomes of trials with negative expected values. Here, participants lost less money after excitatory compared to sham stimulation, whereas participants lost more money in the inhibitory group. Boxplots indicate means (black dots), medians (gray lines) and lower and upper quartiles. Asterisks indicate significance levels: + < 0.1, * < 0.05, ** < 0.01, *** < 0.001.

**FIGURE 5:**
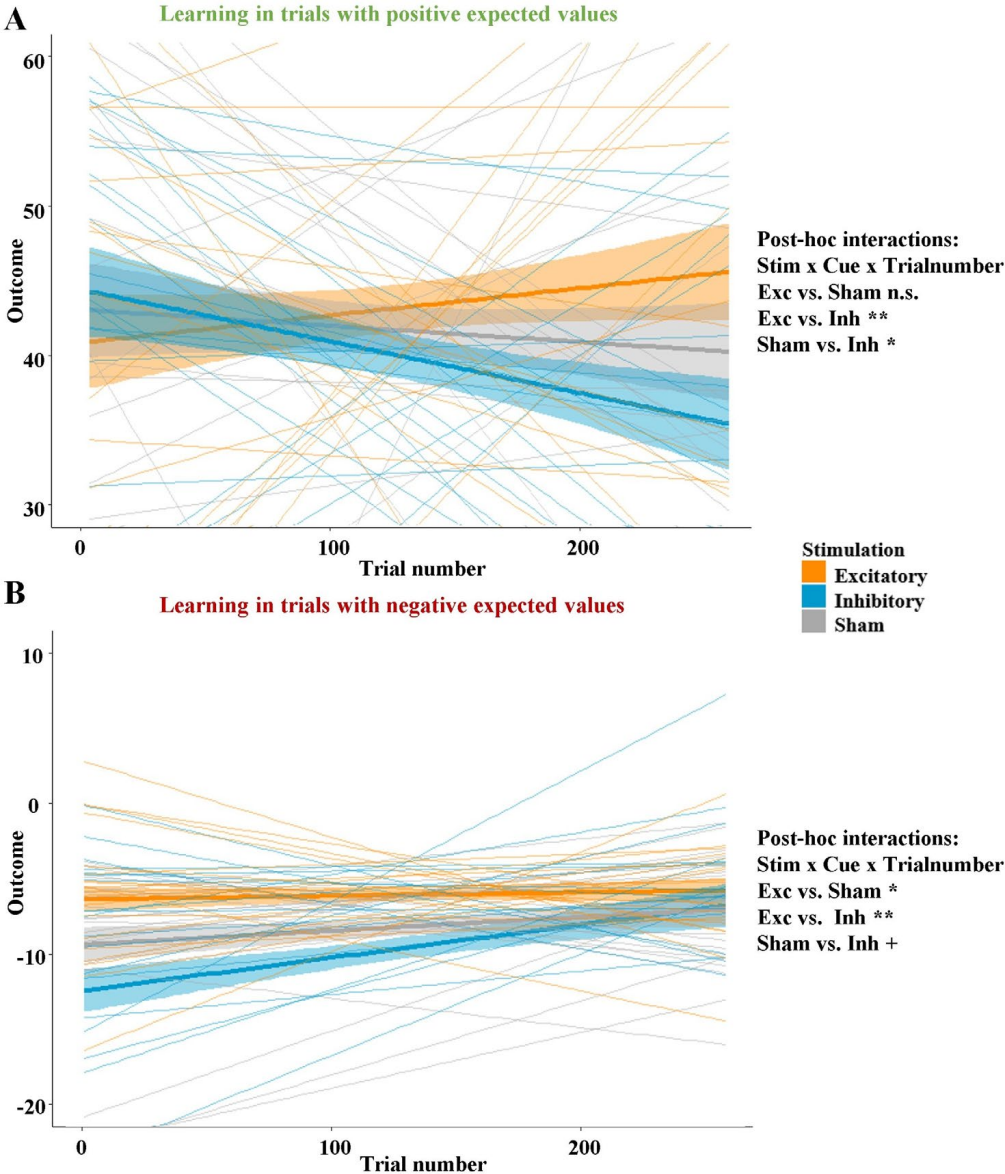
(A) Mean actual outcome per trial as a function of stimulation and trial‐number in trials with positive expected values. This graph shows a pattern consistent with what we have seen in previous studies (Kroker et al. 2025). (B) Mean actual outcome per trial as a function of stimulation and trial‐number in trials with negative expected values. This plot clearly indicates that improved behavioral inhibition is present from the very beginning and that participants in the inhibitory group need longer to learn when to inhibit a high‐risk decision. Importantly, the two conditions show completely opposite patterns and cancel each other out, so that the interaction stimulation × cue × trial number on the risked amount was insignificant. Asterisks indicate significance levels: + < 0.1, * < 0.05, ** < 0.01.

Since we are especially interested in the difference between trials with positive and negative expected values, we tested for main effects of stimulation on the actual outcome in two sub‐analyses. In trials with positive expected values, the actual outcomes did not differ between the stimulation groups (*t* = 0.28, *p* = 0.780; Figure 4A). In the trials with negative expected values, however, stimulation influenced how much money participants lost (*t* = 2.39, *p* = 0.019, partial *η* ^2^ = 0.08; Figure 4B). After excitatory vmPFC‐tDCS, participants lost significantly less money compared to sham stimulation (*t* = 3.10, *p* < 0.001, partial *η* ^2^ = 0.10), while participants in the inhibitory group lost more money than those in the sham group (*t* = −1.75, *p* = 0.047, *partial η* ^2^ = 0.02). Interestingly, the learning pattern looked very different between the two conditions. While both positive (*t* = −2.39 *p* = 0.019, partial *η* ^2^ = 0.08) and negative (*t* = 2.20 *p* = 0.028, partial *η* ^2^ = 0.06) expected value trials showed a significant interaction for stimulation × trial number, the pattern was the exact opposite: In trials with negative expected values, the outcome after excitatory vmPFC‐tDCS was higher from the very beginning compared to sham and inhibitory stimulation, indicating improved behavioral inhibition from the start. In the positive expected value trials, however, this pattern developed over time, indicating improved learning (Figure 5). This was indicated by the interaction of stimulation × expected value × trial number (*t* = 4.58, *p* < 0.001, partial *η* ^2^ = 0.14). At the neural level, we found no main effect of stimulation when looking at the trials with positive and negative expected values within the left dlPFC cluster (*p*‐values > 0.5). However, the correlation between risked amounts and neural activity was present in the trials with negative expected values (*r* = 0.17, *p* = 0.031) but not in trials with positive expected values (*r* = 0.06, *p* = 0.34).

### 3.2 Feedback Phase

#### 3.2.1 Behavioral Effects

The mixed‐effects model using the predictors stimulation (excitatory, inhibitory, sham), reward probability, and actual outcome to analyze the amount bet revealed a significant overall model when compared to the null model (*χ* ^2^(12) = 4962, *p* < 0.001). Here, the interactions of stimulation × outcome (*t* = 2.54, *p* = 0.011, partial *η* ^2^ = 0.02), stimulation × reward probability (*t* = 3.79, *p* < 0.001, partial *η* ^2^ = 0.04), and stimulation × reward probability × outcome (*t* = −3.86, *p* < 0.001, partial *η* ^2^ = 0.05) were all significant. To resolve these interactions, we added another variable to the analysis on an exploratory basis: the probability of the actual outcome occurring. This was done because of one of the vmPFC's cardinal functions: anticipating outcomes and learning from prediction errors. When we looked closer at the three‐way interaction, it became clear that the anticipation of gains and losses had a considerable influence on participants' behavior; therefore, we classified a gain after a high chance of winning (cue 5–7) as expected/likely and a gain after a high risk of losing (cue 1–3) as unexpected/unlikely, and we also followed this logic for losses. This revealed a highly significant three‐way interaction of stimulation × probability of actual outcome × actual outcome (*t* = −4.46, *p* < 0.001, partial *η* ^2^ = 0.08; Figure 5A). The interaction pattern suggests that participants were better able to anticipate likely outcomes after excitatory stimulation, as gains were rated as less positive and losses as less negative. By contrast, unlikely outcomes were rated as more extreme (especially losses) after excitatory compared to sham stimulation, whereas inhibitory vmPFC‐tDCS resulted in the opposite pattern. To verify our impression from this exploratory analysis, we calculated the difference of gains and losses and analyzed this difference with the predictors stimulation and probability of the actual outcome. This revealed the expected interaction of stimulation × probability of actual outcome (*t* = 2.98, *p =* 0.004, *partial η* ^2^ = 0.05; Section 2.2 in Supporting Information).

#### 3.2.2 Neural Effects

**FIGURE 6:**
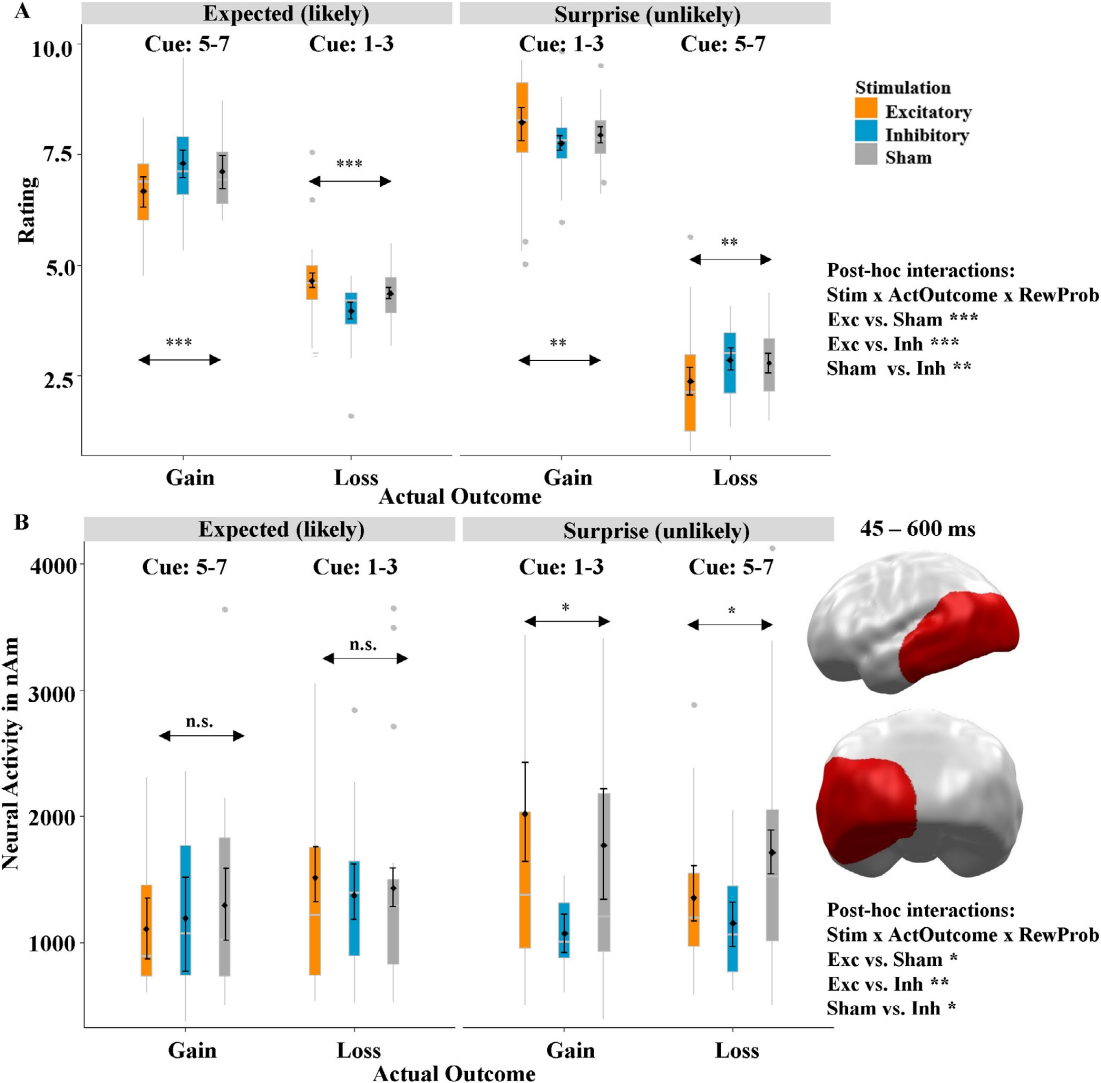
(A) Valence ratings of actual outcome (0: Very negative; 10 very positive) depending on stimulation and reward probability. The presentation of the results was inspired by the neural data, as it is evident that the anticipation/probability of a particular outcome (gain or loss) has a strong influence on its processing. (B) Significant spatiotemporal cluster in left temporal and occipital regions featuring an interaction effect of stimulation × reward probability × outcome. Excitatory stimulation induced an enhanced attention on unexpected gains, while inhibitory stimulation resulted in reduced processing of unexpected outcomes. Asterisks indicate main effects of stimulation with the following significance levels: * < 0.05, ** < 0.01, *** < 0.001.

At the neural level, we observed a significant cluster featuring a three‐way interaction of stimulation × reward probability × outcome (*p*‐cluster = 0.008; Figure 6B) in left occipital, temporal, and parietal areas (temporo‐parietal junction; TPJ). As in the behavioral data, we performed our exploratory approach: Again, the anticipation of outcomes appeared to have an important influence on the activity in this cluster, since there was no interaction effect of stimulation × actual outcome after expected/likely outcomes (*t* = 0.026, *p* = 0.920). The interaction of stimulation × actual outcome after unexpected/unlikely outcomes, however, drove the three‐way interaction, as here a significant interaction occurred (*t* = 1.81, *p* = 0.044, partial *η* ^2^ = 0.11). Driven by the exploratory analysis in the behavioral data, we calculated the difference between gains and losses, which likewise showed an interaction of stimulation × probability of actual outcome (*t* = 2.21, *p =* 0.034, partial *η* ^2^ = 0.03; Section 2.2 in Supporting Information). Contrary to the behavioral interaction, this effect was only driven by the main effect of stimulation after unexpected/unlikely outcomes (*t* = 1.98, *p =* 0.052, partial *η* ^2^ = 0.05), while the main effect after expected/likely outcomes was insignificant (*t* = −0.74, *p =* 0.44). Interestingly, the neural activity in this cluster was correlated to the difference (gain–loss) in the feedback ratings (*r* = 0.18, *p* = 0.043). This correlation was strongly driven by the excitatory condition (*r* = 0.42, *p* = 0.007), while the respective correlations were insignificant in the sham (*r* = −0.16, *p* = 0.296) and inhibitory conditions (*r* = 0.09, *p* = 0.575).

## 4 Discussion

In the present paper, we show that excitatory vmPFC‐tDCS improves decision‐making in positive and negative expected value trials, while inhibitory stimulation impaired gambling behavior compared to the sham group. Particularly, excitatory vmPFC‐tDCS improved behavioral inhibition, resulting in superior outcomes after excitatory stimulation in negative expected value trials. The neural results indicate that excitatory vmPFC‐tDCS enhanced participants' ability to inhibit incautious behavioral approach behavior when the risk of losing was high. They also highlight that the interplay between ventral and dorsal prefrontal brain regions is important in decision‐making. The behavioral data from the feedback phase suggest that excitatory vmPFC‐tDCS led participants to better anticipate the likely outcomes, since they gave less extreme ratings after likely outcomes, and an enhanced processing depth when facing unexpected outcomes, as they gave more extreme ratings after unlikely outcomes. At the neural level, vmPFC‐tDCS modulated feedback processing as a function of outcome probability. The processing of unlikely gains seemed to be especially enhanced after vmPFC excitation, while inhibitory stimulation appeared to impair the processing of unexpected outcomes compared to sham stimulation.

Our results demonstrate that vmPFC‐targeted stimulation can modulate and improve decision‐making through allowing participants to better assess their chances and risks. This was reflected in the excitatory group's behavior of betting higher amounts when the chance of winning was high and smaller amounts when the risk of losing was high. However, this improved gambling behavior did not result in better overall outcomes. In contrast to the excitatory group, the inhibitory group showed a particularly impaired ability to assess risks, leading to unreasonable high‐risk decisions (Figure 3A). However, convergent with our previous studies (Kroker et al. 2025), this comparison was only significant between excitatory and inhibitory stimulation, while the inhibitory group did not differ from sham stimulation, which was present in our previous studies (Kroker et al. 2025).

The underlying mechanisms for the excitatory group's better gambling behavior appear to be improved learning from gains, as this effect developed over the course of the paradigm (Figure 4A), and improved behavioral inhibition, which was present from the beginning of the experiment (Figure 4B). This finding can be better interpreted in light of the feedback data, which suggest that the processing depth of outcomes is enhanced following vmPFC excitation, such that participants were better able to anticipate outcomes in the following trials. This aligns with our previous research (Kroker et al. 2022, 2025; Kroker, Rehbein, et al. 2023; Kroker, Wyczesany, et al. 2023) and results from other groups (Lapenta et al. 2018; Ouellet et al. 2015; Vöckel et al. 2024), indicating that vmPFC excitation can improve decision‐making and behavioral inhibition. Furthermore, this result fits findings showing that patients with vmPFC lesions perform worse on gambling tasks (Bechara, Tranel, and Damasio 2000; Reber et al. 2017).

At the neural level, we observed a cluster in the left dlPFC and parietal regions in mid‐latency to late time intervals showing an interaction of stimulation and cue. It is important to note that the post hoc tests were inconclusive, as the effect of excitatory stimulation was marginally significant, while the effect of inhibitory stimulation was not significant. Only the excitatory and inhibitory groups differed significantly. The neural activity in this cluster was correlated with the amount bet, indicating an association of this cluster with reward approach behavior. Interestingly, this correlation was only present in the inhibitory and sham groups, suggesting that excitatory vmPFC stimulation might inhibit unreasonable approach behavior. This is also supported by the fact that the correlation between the amount bet and left parietal/dlPFC activity was only significant in the trials with negative expected values. The evidence for this finding is mixed, as some studies suggest that the right dlPFC is more involved in behavioral inhibition while the left is rather associated with behavioral approach (Knoch et al. 2006). Yet, consistent with our findings, one study found reduced left dlPFC activity in patients with depression (Bruder et al. 2017), who show reduced approach behavior. At the same time, another study showed that excitatory rTMS of the left dlPFC had a strong effect on reducing cravings but smaller effects for other neuropsychiatric disorders (Kan et al. 2023). Overall, this may mean that the assessment of chances/risks and the actual making of decisions is likely processed in a variety of brain regions and might be governed by the vmPFC and dlPFC.

Another important aspect to consider is the anticorrelation of vmPFC and dlPFC activity, since the vmPFC is part of the default mode network and the dlPFC is part of the task‐positive network (Cheng et al. 2020; Smallwood et al. 2021). Other research has suggested that decision‐making involves anticipatory gamma and theta activity in the vmPFC, when expecting a stimulus, in combination with dorsal PFC theta activity when the stimulus actually occurs (Adelhöfer and Beste 2020; Domenech et al. 2020). Thus, the default mode activity of the vmPFC appears to be associated with the preparation of behavioral inhibition (Adelhöfer and Beste 2020) as well as the anticipatory value encoding of a stimulus, and the dorsal PFC is then involved in the actual execution of a strategy (Domenech et al. 2020). This may be why both stimulation targets could be effective in reducing the symptoms of behavioral addictions, such as poor behavioral inhibition and craving. However, they may work via different (learning) mechanisms, as described later. Future research should therefore determine which groups of patients would benefit more from dlPFC or vmPFC stimulation.

Since we were especially interested in the difference between positive and negative expected value trials, we performed separate analyses on the actual outcomes for both conditions. This revealed no modulation of outcomes in the positive expected value trials, but in the negative expected value trials the excitatory group lost significantly less money compared to the sham group, whereas the inhibitory stimulation group had greater losses (Figure 4). This improvement in behavioral inhibition makes excitatory vmPFC‐tDCS particularly interesting as a therapeutic option for disordered gambling and other behavioral addictions. Particularly, a combination with cue‐exposure therapy is promising, as previous results have shown an enhanced efficacy of extinction learning in fear paradigms after vmPFC excitation (Dittert et al. 2018; Van't Wout et al. 2016; Vicario et al. 2020).

Importantly, the learning mechanism between the stimulation groups in the positive and negative expected value trials was completely inverted (Figure 5). Learning in the positive expected value trials showed the well‐known pattern that we observed in previous studies (Kroker et al. 2022; Kroker, Wyczesany, et al. 2023). In these trials, all stimulation groups started at a similar level, and the excitatory group improved while the inhibitory group worsened over time compared to the sham stimulation group. By contrast, in the trials with negative expected values, excitatory stimulation induced superior gambling behavior (i.e., behavioral inhibition) from the very beginning, and the inhibitory group performed worse than the sham group. Ultimately, this suggests that excitatory stimulation could help patients to regain behavioral inhibition, while the inhibitory group could serve as a scientific model for the neural mechanisms of behavioral (Perales et al. 2020), specifically because the inhibitory group took longer to learn when to inhibit an irrational behavioral response (such as betting a large amount of money). This leads to the conclusion that learning from gains/rewards is different from learning from losses/punishments. Furthermore, the learning mechanisms after vmPFC and dlPFC stimulation may be different, and further studies on these learning mechanisms would help researchers understand why both stimulation targets seem to be effective. To this end, a closer look at the feedback data also seems helpful to understand how vmPFC stimulation modulates learning from gains and losses.

In the feedback phase, we observed a modulation of behavioral and neural responses by the stimulation as a function of the probability of an outcome. Our exploratory analysis clearly showed that following excitatory stimulation, expected or likely gains were rated less positively and losses were rated less negatively, suggesting that excitatory stimulation helped participants better anticipate outcomes (compared to those in the sham stimulation group). Unexpected or unlikely outcomes, on the other hand, were rated as more extreme, indicating that excitatory stimulation allowed participants to more deeply process feedback stimuli and more accurately update their predictions after unexpected outcomes. The improvements in these two key abilities following excitatory stimulation may help patients learn more efficiently from the feedback they receive. This, in turn, may allow them to more quickly disengage from maladaptive behaviors, which is typically difficult for patients with vmPFC lesions or behavioral addictions, which are associated with vmPFC hypoactivity (Bechara, Tranel, and Damasio 2000; van Holst et al. 2010a). Again, inhibitory stimulation induced deteriorated gambling compared to the sham group and a pattern resembling the behavior of people with behavioral addictions.

At the neural level, we observed a similar pattern as that found in the behavioral data in left occipital and temporo‐parietal (TPJ) areas: We observed an enhanced processing of unexpected outcomes following excitatory and sham stimulation compared to inhibitory stimulation. We interpret this as an effect of the governing function of the vmPFC, since it has a strong connectivity to the TPJ (Murray et al. 2015), a region associated with goal‐directed attention (Corbetta and Shulman 2002; Smith et al. 2014). The interaction of the vmPFC and the TPJ appears to control attention in reward processing (Smith et al. 2014), particularly when an unexpected event occurs—a so‐called prediction error. In the case of expected outcomes, no further attentional processing is required in the TPJ, so this condition was not modulated by the stimulation. However, in the case of unexpected or salient feedback, the TPJ becomes active to process stimuli that are particularly relevant to attention (Corbetta and Shulman 2002). This process can even be modulated with TPJ stimulation (Radecke et al. 2023).

Interestingly, exploring the processing of unlikely outcomes revealed that vmPFC excitation especially enhanced the attention on unexpected gains compared to the inhibitory stimulation, suggesting different learning from gains and losses. This is further underlined by the positive correlation between the activity in this cluster and the difference between gains and losses in the behavioral ratings. Importantly, this correlation was primarily driven by the excitatory and unexpected/unlikely conditions, suggesting that excitatory vmPFC stimulation enhances the attentional processing of unexpected outcomes (i.e., prediction errors), from which better predictions are derived in the long‐term. We observed a similar pattern in a previous study, where vmPFC stimulation primarily modulated the gain prediction error and (but to a lesser extent) the loss prediction error (Rehbein et al. 2023), which makes sense because the vmPFC is also part of the reward system (Arias‐Carrián et al. 2010). This may partly explain the different learning patterns in positive and negative expected value trials between the stimulation groups (Figure 5). Improved learning from gains and reward prediction errors following excitatory stimulation needs to develop over time, while improved behavioral inhibition represents another vmPFC function. Such a versatile function of the vmPFC has been proposed in previous work (Hiser and Koenigs 2018).

### 4.1 Limitations and Implications for Future Research

Overall, our study provides novel insights into the potential causal role of the vmPFC in decision‐making during gambling, feedback processing, and learning. Nevertheless, several aspects and limitations warrant further consideration. First, we investigated the effects of vmPFC stimulation in healthy participants to see whether positive results indicate clinical benefit in patients with altered vmPFC function. However, our results don't allow conclusions about the clinical use of excitatory vmPFC‐tDCS in patients with disordered gambling or other behavioral addictions. Future studies will need to demonstrate that the effects of stimulation generalize to patients. Second, we must acknowledge that tDCS has limited focality (Gross et al. 2023) and our stimulation montage also co‐stimulated, though to a smaller degree, other prefrontal regions, such as the dmPFC, implicated in decision‐making and learning. To solve this issue, other stimulation methods, such as rTMS (repetitive Transcranial Magnetic Stimulation), could be used to stimulate the ventral prefrontal target regions more precisely. However, TMS often induces unpleasant co‐stimulation of eye and facial muscles, in particular when applied in ventral PFC target regions (Overvliet et al. 2021). As inhibitory and excitatory rTMS use different stimulation paradigms, these undesirable aversive perceptions of the stimulation would differ between stimulation conditions, which would make it considerably more difficult to balance and blind the experimental groups. Thus, we decided to opt for tDCS as it allows excellent blinding with a reasonable focality (Figure 2). As structural imaging data of our novice participants were not available and to allow better comparisons and tests of replicability with our previous studies, we used the standard stimulation procedure which has been developed based on cubic finite element modeling (Wagner et al. 2014). Individualized vmPFC targeting, using state‐of‐the‐art FEM based software packages like SimBio (Wagner et al. 2014), DuneNeuro (Schrader et al. 2021), SimNIBS (Thielscher et al. 2015) or others, might provide a more individualized stimulation in future studies.

Third, we did not find an interaction effect with stimulation that overlapped with the vmPFC, which was surprising since this was our stimulation target. A potential explanation for this lack of effect could be that modulated vmPFC excitability also exerts its effects on postsynaptic neurons (Farahani et al. 2021). Furthermore, it is a common finding that stimulation effects occur in remote but not in target regions (Angelini et al. 2024; Paulk et al. 2022). Fourth, we must acknowledge that interindividual differences in risk tolerance and decision‐making have a greater limiting effect in the design chosen here between subjects than in designs within subjects. To address this, we gathered relevant questionnaires (REI‐40, RR, and UI‐18) and investigated whether there were group differences between the stimulation conditions. By showing that no differences exist between the groups, we aimed to minimize the influence of individual differences on our results. The main reason to employ a between‐subjects design was that we wanted to include a sham condition, as subjects in a within‐subjects design would typically notice the difference between active and sham (placebo) stimulation. Finally, our findings of vmPFC excitation on decision‐making should not be generalized to imply that healthy participants always perform better after vmPFC stimulation. Healthy participants with normal vmPFC excitability may show adverse effects after vmPFC stimulation in other mental tasks where lower vmPFC excitability would better serve their goals. Future studies should address these limitations by investigating which group of participants/patients would rather profit from tDCS or rTMS and from vmPFC or dlPFC stimulation.

## 5 Conclusion

In summary, we found with high reliability that vmPFC excitation can improve decision‐making and feedback processing in gambling, while inhibitory stimulation impairs these processes compared to sham stimulation. This is true for positive and negative expected value trials, although the respective learning mechanisms are completely inverted; this suggests different learning from gains and losses. As such, excitatory vmPFC‐tDCS might be a promising add‐on treatment option for disordered gambling and other behavioral addictions, particularly in combination with cue‐exposure therapy. Thus, vmPFC‐tDCS may make it easier for patients to disengage from their maladaptive, dysfunctional, and time‐consuming behaviors by improving behavioral inhibition, increasing processing depth, and anticipating outcomes over time, i.e., facilitating learning to regain control over their behavior (Perales et al. 2020).

## Author Contributions

**Thomas Kroker:** conceptualization, methodology, formal analysis, writing – review and editing, writing – original draft, visualization. **Maimu Alissa Rehbein:** conceptualization, writing – review and editing. **Miroslaw Wyczesany:** writing – review and editing. **Riccardo Bianco:** writing – review and editing. **Alejandro Espino‐Paya:** writing – review and editing. **Markus Junghöfer:** conceptualization, writing – original draft, writing – review and editing, visualization, methodology, software, funding acquisition, supervision.

## Funding

This work was supported by Deutsche Forschungsgemeinschaft (JU 445/9‐1), Narodowe Centrum Nauki (UMO‐2018/31/G/HS6/02490), Interdisciplinary Center for Clinical Research of the University of Muenster (Ju2/024/15).

## Conflicts of Interest

The authors declare no conflicts of interest.

## Supporting information


**Data S1:** psyp70227‐sup‐0001‐supinfo1.docx.

## Data Availability

The data that support the findings of this study are available from the corresponding author upon reasonable request.
