# Supplementary material for: Non‐Invasive Brain Stimulation of the Ventromedial Prefrontal Cortex Improves Behavioral Inhibition by Enhancing the Processing Depth and Anticipation of Outcomes in a Gambling Task
Source: Psychophysiology. 2026 Jan 9;63(1):e70227. doi: 10.1111/psyp.70227 (PMC12789285; doi:10.1111/psyp.70227)
Supplement: Supplementary file 1 — Data S1: psyp70227‐sup‐0001‐supinfo1.docx. [file PSYP-63-e70227-s001.docx]

**Supplementary Materials**

**Non-invasive brain stimulation of the ventromedial prefrontal cortex improves behavioral inhibition by enhancing the processing depth and anticipation of outcomes in a gambling task**

**Thomas Kroker, PhD^1,2^, Maimu Alissa Rehbein, PhD^1,2,4^, Miroslaw Wyczesany, PhD^3^, Riccardo Bianco, MSc^1,2^, Alejandro Espino-Paya, PhD^1,2^ & Markus Junghöfer, PhD^1,2^**

^1^ Institute for Biomagnetism and Biosignalanalysis, University of Muenster, Germany

^2^ Otto Creutzfeldt Center for Cognitive and Behavioral Neuroscience, University of Muenster, Muenster, Germany

^3^ Institute of Psychology, Jagiellonian University, Krakow, Poland

^4^ Institute of Psychology, Unit of Clinical Psychology and Psychotherapy for Children and Adolescents, University of Osnabrueck

Corresponding author:

Thomas Kroker

Institute for Biomagnetism and Biosignalanalysis

University of Muenster

Malmedyweg 15

48149 Muenster, Germany

Email: thomas.kroker@uni-muenster.de

# **Methods**

## **1.1. Participants**

Exclusion criteria, were current or lifetime psychiatric diagnosis, psychopharmaco­logical treatment, current or past psychotherapy, neurological or severe somatic illness, pregnancy and prior participation in a gambling study. The latter would result in knowledge about our cover story. Participants were recruited from the existing participant pools of our institute, announcements on the university campus and via social media.

## **1.2. tDCS**

All participants received one tDCS condition (anodal/excitatory, cathodal/inhibitory, or sham/placebo) before the gambling task. To ensure blinding, the stimulation device was positioned outside the participants’ field of view, and the study was conducted using the double-blind mode of the neuroConn DC-STIMULATOR, which allows condition-specific stimulation protocols (active or sham) to be pre-programmed. These codes were entered by the experimenter, who remained unaware of the assigned stimulation condition throughout the session. In the anodal condition, stimulation is presumed to cause a relative depolarization of neuronal membrane potentials, thereby increasing cortical excitability. In contrast, cathodal stimulation is associated with hyperpolarization, reducing excitability (Polanía et al., 2018). For all conditions, stimulation was delivered via saline-soaked sponge electrodes to ensure effective conductivity. A 10-second ramp-up (fade-in) and 10-second ramp-down (fade-out) were used to avoid abrupt sensory perceptions. In the anodal and cathodal conditions, a 1.5 mA current was applied for 10 minutes. In the sham condition, stimulation lasted only for 30 seconds before being discontinued, though the device display and session duration were identical across conditions to preserve blinding.

The study employed a between-subjects design to facilitate effective sham control. In within-subject designs—especially when using small electrodes with higher current densities, as in this study—participants may more easily detect stimulation differences.

## **1.3. Recording and Preprocessing of EEG Data**

We performed EEG measurements using a 128-channel system (EGI systems, Oregon, USA), which measured frequencies between 0 and 150 Hz at a sampling rate of 1000 Hz. The data were sampled at a rate of 500 Hz and filtered with a 0.1 Hz high pass filter and a 48 Hz low pass filter. Epochs from 200 ms before to 600 ms after stimulus onset were extracted and baseline-adjusted using the -150 ms to 0 ms pre-stimulus interval. For statistical correction and artifact rejection, the method proposed by Junghöfer and colleagues (2000) was used. This procedure identifies artifacts of individual channels as well as global artifacts. If noisy channels are detected, their signal is estimated by spherical-spline interpolation based on the weighted signal of all remaining sensors. A minimum threshold of 0.01 was used for the estimated goodness of interpolation, and trials exceeding this value were rejected. If more than 30% of the trials in the EEG baseline or test phase were rejected, the participant was excluded from further analysis. Nine participants were excluded due to global cross-conditional EEG artifacts (excitatory: N = 3, inhibitory = 4, sham = 2).

The estimation of underlying neuronal sources was performed using the L2-Minimum Norm Estimate (L2-MNE) inverse modeling technique, which is a common approach in MEG and EEG analysis (Hämäläinen & Ilmoniemi, 1994). The source model employed a spherical head model containing 350 evenly distributed dipole pairs located on a shell corresponding to the estimated gray matter depth (87% of the individually fitted head radius). Source topographies were established using a Tikhonov regularization parameter of k=0.1, and the final output was the source-direction-independent neural activity (the vector length of the estimated activity) for each participant, condition, and time point.

A significant challenge with inverse modeling, known as the inverse problem, is the inability to distinguish between deep, focal sources and superficial, distributed sources, as both can produce similar surface signals. While applying L2-Minimum-Norm depth weighting with realistic head models can attenuate this bias towards superficial sources, this step was omitted because individual MRIs for realistic source modeling were not available. Consequently, the estimated activity of deeper neural structures is likely projected onto the surface and superposed with activity arising from the estimated superficial gray matter. Additionally, individual noise covariance estimation, which could have improved source reconstruction precision, was not applied.

## **1.4. Statistical Analysis of EEG Data**

To address the pervasive multiple comparisons problem across both time points and source locations, we employed the cluster-based permutation test proposed by (Maris and Oostenveld (2007). This method first calculates the statistical value at every estimated neural source (dipole) and time point, testing for an initial significance at a sensor-level criterion of p=0.01. Adjacent time points and sources that exceed this threshold are then grouped into a single spatio-temporal cluster, and the cluster's overall magnitude, or cluster-mass, is calculated from the combined statistical value, spatial extent, and temporal extent.

In the second and critical step, the observed cluster masses within pre-defined time intervals (early: 0–300 ms; late: 300–600 ms) were tested against a null distribution constructed from the cluster masses of 1,000 permuted drawings of the same data set. If the observed cluster-mass exceeded the critical cluster-mass corresponding to the cluster-level criterion of *p* = 0.05 (i.e., greater than 95% of the permuted clusters), the entire spatio-temporal effect was classified as significant. This robust procedure was systematically applied to all examined effects.

# **Results and discussion**

## **2.1. Cue-Phase**

**Behavioral Effects**

The mixed-effects-model using the predictors stimulation (excitatory, inhibitory, sham), cue (1-7), and trial number (1-256) revealed a significant main effect of stimulation (*t* = -2.93, *p* = 0.005*, η2* = 0.15; see Fig. SM1). Post-hoc tests show no difference between excitatory and sham stimulation (*t* = -0.11, *p* = 0.916), while the effects between excitatory and inhibitory stimulation (*t* = -4.25, *p* < 0.001*, η2* = 0.03) as well as inhibitory and sham stimulation were significant (*t* = -4.23, *p* < 0.001*, η2* = 0.03).

This effect again underlines the idea that hypoactivation of the vmPFC results in unreasonable or maladaptive behavior, which is typical for behavioral addictions (Antons et al., 2020; Perales et al., 2020; van Holst et al., 2010; Van Holst et al., 2010). Additionally, it again suggests that inhibitory vmPFC stimulation might serve as a neurobiological model for pathological gambling or other behavioral addictions, even if this model is probably not quite complete.


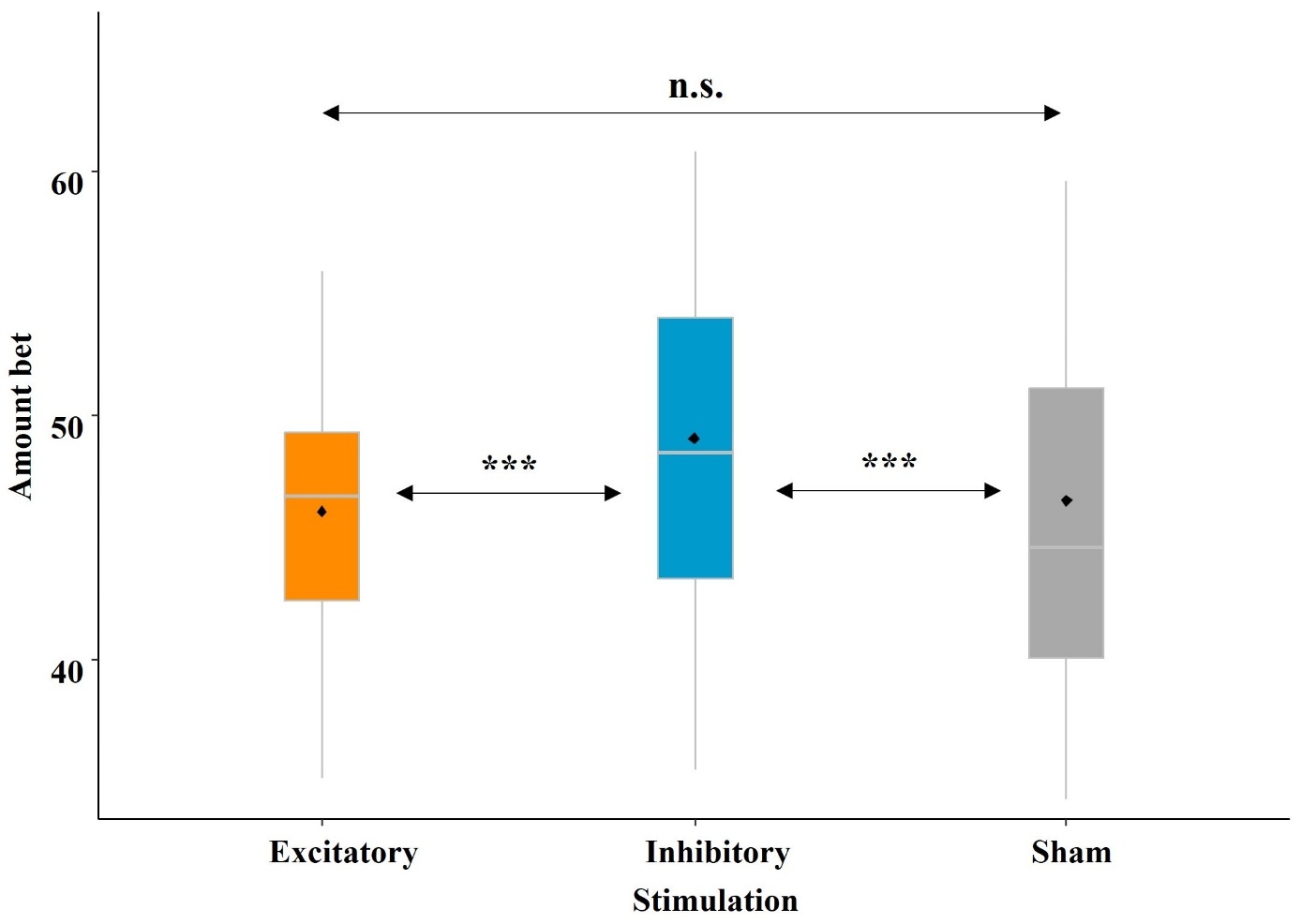
*Figure SM1.*

Risked amounts in dependency of the stimulation. More money is risked in the inhibitory group compared to the excitatory and sham group.

**Neural main effects**

At the neural level, we observed a main effect of cue covering the majority of the brain (*p*-cluster < .001) and the complete time interval (0-600 ms). Descriptively, neural activity was higher in response to ambiguous cues (cues 3-5) compared to the cues with relatively clear gain/loss probabilities (cues 1, 2, 6, 7). Thus, we tested the respective assumption statistically, which revealed a strong main effect for distinct versus ambiguous cues (*t* = 4.67, *p* < 0.001*, η2* = 0.27).

The observed cluster might be due arousal effects spreading across the whole brain. Ambiguous cues might be more arousing compared to relatively distinct cues, which has already been suggested in previous publications (Zheng et al., 2020). However, due to the large temporal and spatial extent of this cluster its interpretation is challenging.


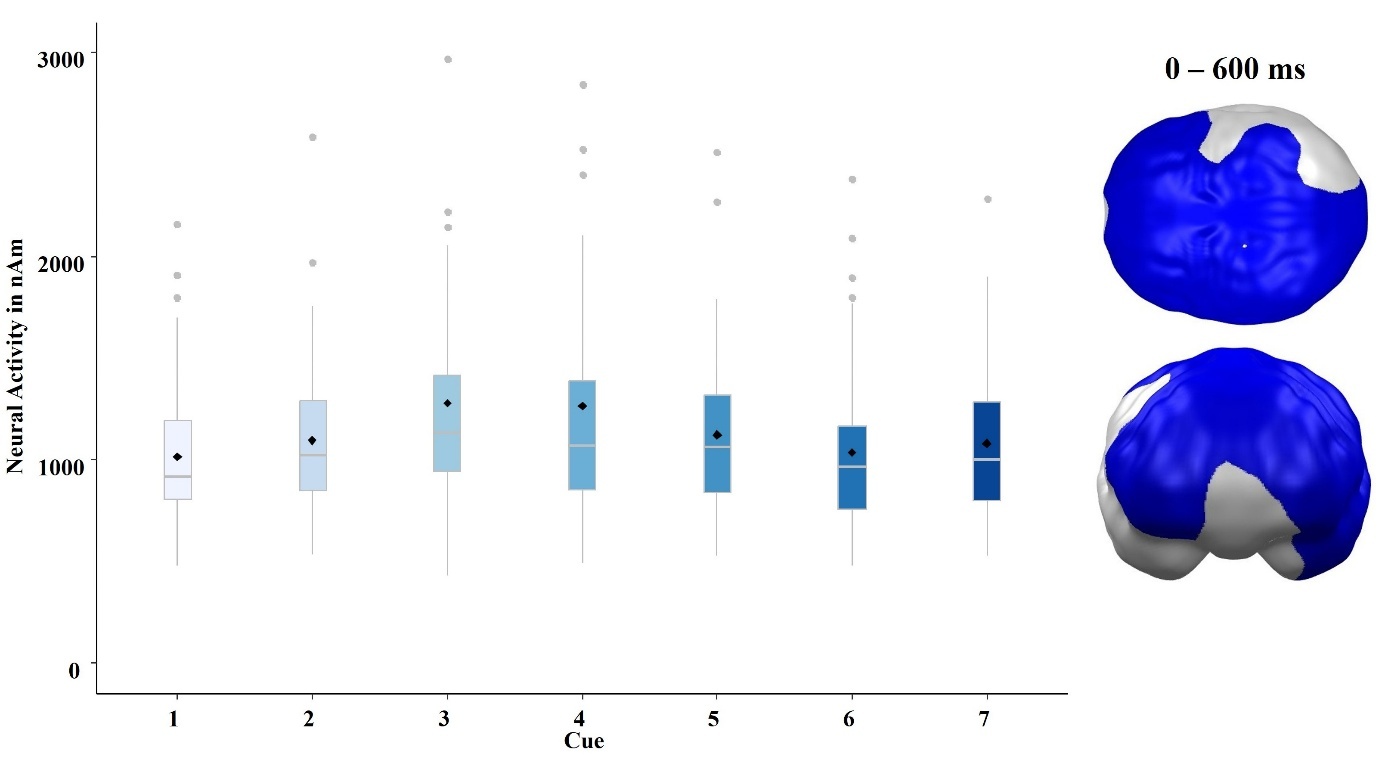
*Figure SM2.*

Neural main effect in response to cue in the cue phase indicate a greater activation in response to more ambiguous cues, while the cues, which signal a clearer reward probability elicit less neural activity.

## **2.2. Feedback-Phase**

**Neural main effects**

This main effect of the risk of losing or chance of winning respectively again covers almost the whole brain and the complete time interval (*p*-cluster < .001; see Fig. SM3). This cluster shows some parallels to the cluster shown above (Fig. SM2) and are most probably explained by arousal effects driven by loss aversion, i.e. the negative evaluation of losing is twice as strong (arousing) as the positive evaluation of an identical win (Kahneman & Tversky, 1979). Thus, high-risk of losing cues evoke more arousal in the brain than cues that offer a high chance of winning.


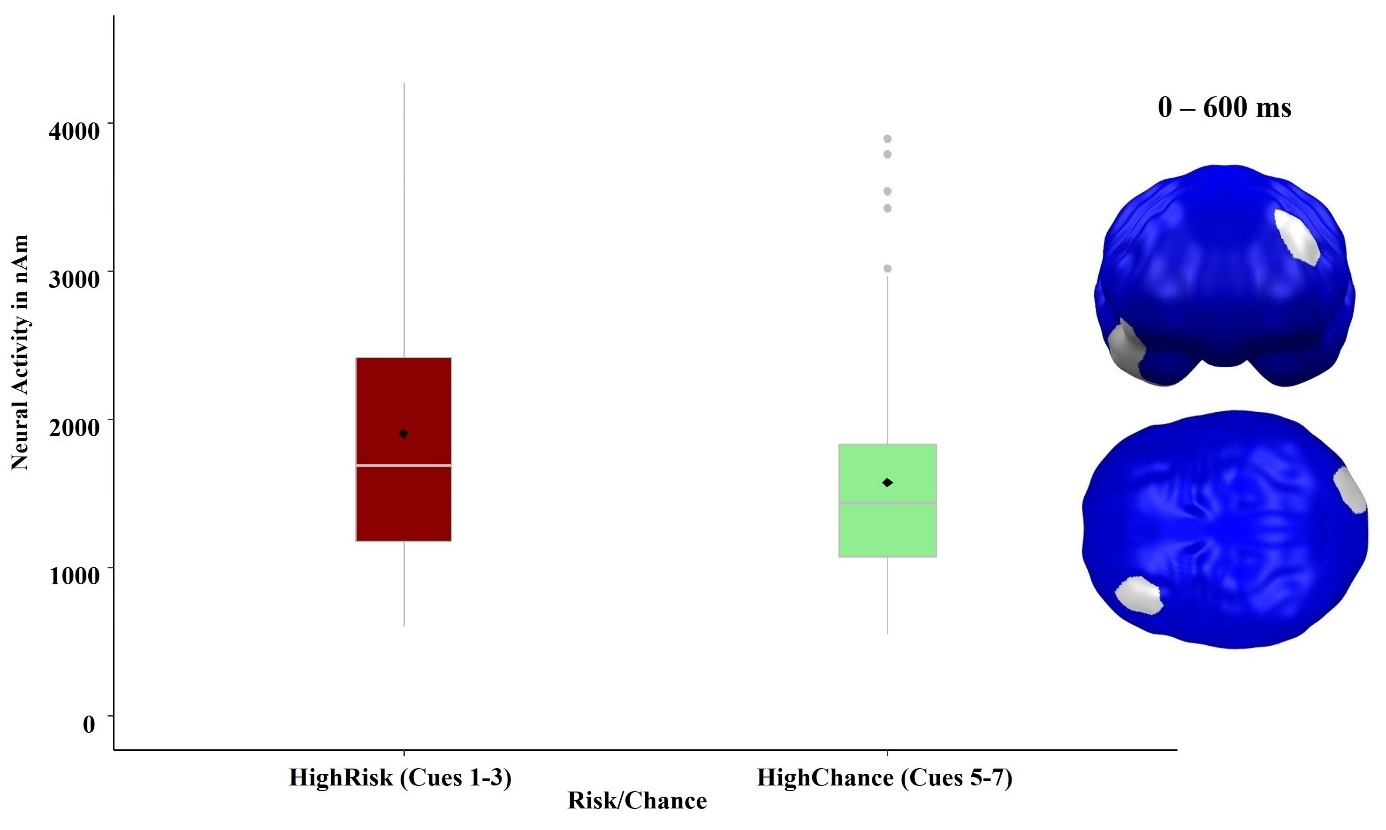
*Figure SM3.*

Neural main effect in response to the risk in the feedback phase. This indicates a greater activation in response to higher risk of losing (i.e., cues 1-3) compared to cues with a higher chance of winning (i.e., cues 5-7).

**Analysis on the difference of gain minus loss**

To further clarify the interaction of stimulation, reward probability and outcome reported in the main text (see Fig. 6 main text, we here depict the difference between gains and losses. At the behavioral level, we analyzed the resulting differences with the predictors stimulation and probability of the actual outcome which revealed a significant main effect of the probability of the actual outcome (*t* = 3.08, *p <* 0.001, *η2* = 0.14) and the interaction of stimulation × probability of actual outcome (*t* = 2.98, *p =* 0.004, *η2* = 0.05). The main effect of probability of the actual outcome indicates that the difference between unexpected/unlikely gains and losses was greater. In fact, an expected gain or loss should be experienced less intensely, and therefore ratings should be less extreme. Importantly, this effect is enhanced by excitatory stimulation as the interaction of stimulation × probability of actual outcome indicates. This interaction suggests that the anticipation of expected/likely outcomes and the enhanced processing depth of unexpected/unlikely outcomes plays an important role: the difference between expected/likely gains and losses is smaller after excitatory stimulation, indicating that the outcome has already been anticipated, resulting in a less extreme rating. After unexpected/unlikely losses on the other hand, the difference is bigger, suggesting an enhanced processing depth, when a behavioral adaption is necessary after an unexpected outcome (Hiser & Koenigs, 2018).

The analysis on the neural differences (see Fig. 6 main text and Fig. SM4) showed a main effect of the probability of the actual outcome (*t* = 3.01, *p <* 0.001, *η2* = 0.05) and an interaction effect of stimulation by probability of the actual outcome (*t* = 2.98, *p =* 0.004, *η2* = 0.05). The main effect of the probability of the actual outcome indicates greater activity in response to unexpected/unlikely gains (minus losses) compared to expected/likely outcomes. The interaction effect suggests that this tendency is further enhanced by excitatory stimulation. Furthermore, the interaction effect is almost entirely driven by the main effect of stimulation after unexpected/unlikely outcomes (*t* = 1.98, *p =* 0.052, *η2* = 0.05), while the respective effect was insignificant after expected/likely outcomes (*t* = -0.74, *p =* 0.44). This indicates that excitatory stimulation could enhance the attentional processing of unexpected/unlikely outcomes in the TPJ and adjacent areas (Corbetta & Shulman, 2002; Smith et al., 2014). Thereby, especially the processing of unexpected/unlikely gains seems to enhanced.


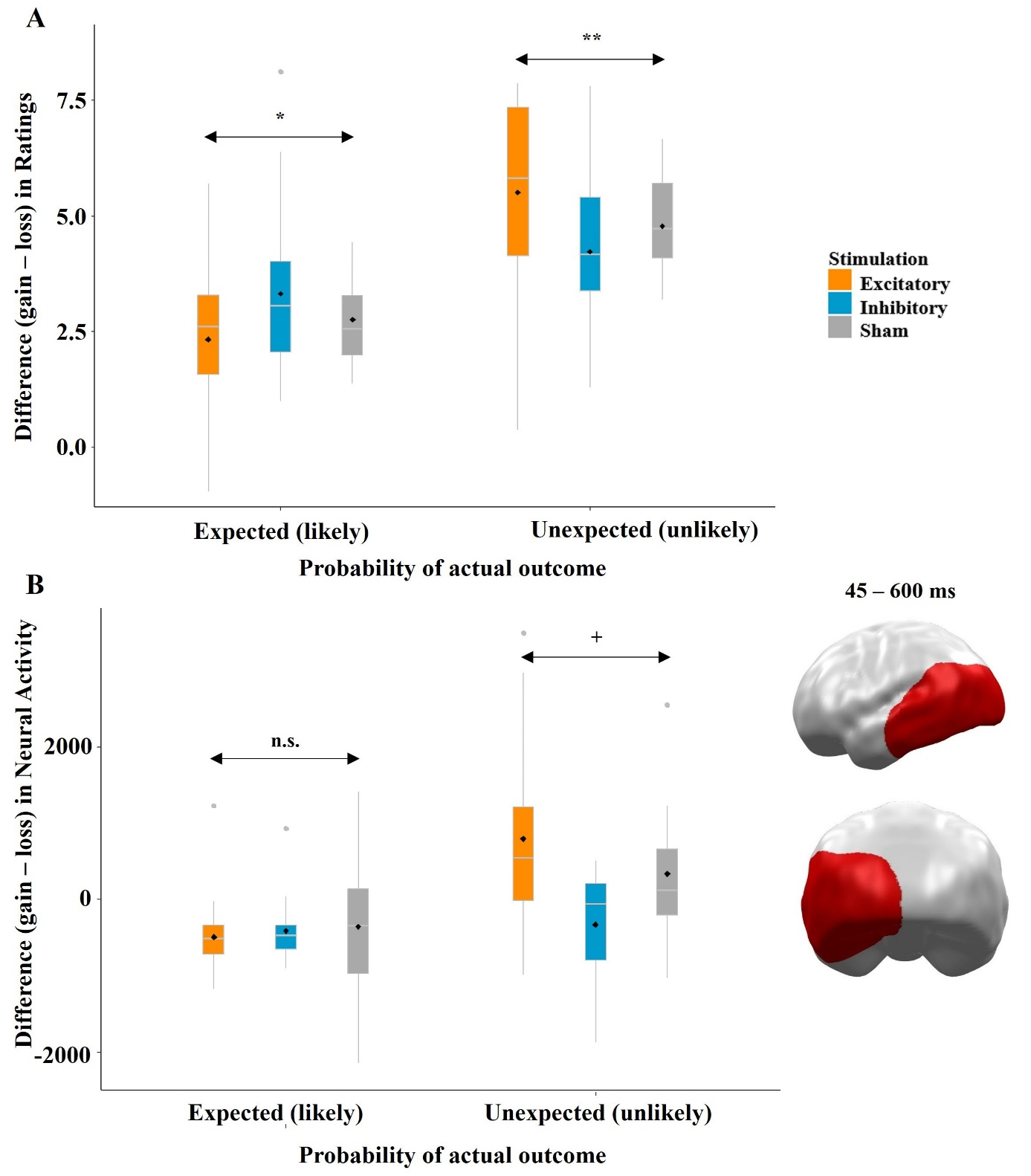
*Figure SM4.*

**A.** Difference (gain-loss) in ratings in dependency of stimulation and reward probability. This clearly indicates a smaller difference in the excitatory group when the outcomes was expected compared to sham and inhibitory stimulation, while expected outcomes elicit the opposite pattern.

**B.** Difference in neural activity (gain-loss) in in dependency of stimulation and reward probability. Here, we see the opposite pattern to the behavioral data, possibly indicating attentional processes.

Asterisks indicate main effects of stimulation with the significance levels: + < 0.1, * < 0.05, ** < 0.01, *** < 0.001.

**References**

Antons, S., Brand, M., & Potenza, M. N. (2020). Neurobiology of cue-reactivity, craving, and inhibitory control in non-substance addictive behaviors. In *Journal of the Neurological Sciences* (Vol. 415). Elsevier B.V. https://doi.org/10.1016/j.jns.2020.116952

Corbetta, M., & Shulman, G. L. (2002). Control of goal-directed and stimulus-driven attention in the brain. *Nature Reviews Neuroscience*, *3*(3), 201–215. https://doi.org/10.1038/nrn755

Hämäläinen, M., & Ilmoniemi, R. (1994). Interpreting magnetic fields of the brain: minimum norm estimates. *Medical & Biological Engineering & Computing*, *32*(1), 35–42. https://doi.org/10.1007/BF02512476

Hiser, J., & Koenigs, M. (2018). The multifaceted role of the ventromedial prefrontal cortex in emotion, decision-making, social cognition, and psychopathology. *Biological Psychiatry*, *83*(8), 638–647. https://doi.org/10.1016/j.biopsych.2017.10.030.

Junghöfer, M., Elbert, T., Tucker, D. M., & Rockstroh, B. (2000). Statistical control of artifacts in dense array EEG/MEG studies. *Psychophysiology*, *37*(4), 523–532. https://doi.org/10.1017/S0048577200980624

Kahneman, D., & Tversky, A. (1979). Prospect Theory: An Analysis of Decision under Risk. *Econometrica*, *47*(2), 263–291. https://econpapers.repec.org/RePEc:ecm:emetrp:v:47:y:1979:i:2:p:263-91

Maris, E., & Oostenveld, R. (2007). Nonparametric statistical testing of EEG- and MEG-data. *Journal of Neuroscience Methods*, *164*(1), 177–190. https://doi.org/https://doi.org/10.1016/j.jneumeth.2007.03.024

Perales, J. C., King, D. L., Navas, J. F., Schimmenti, A., Sescousse, G., Starcevic, V., van Holst, R. J., & Billieux, J. (2020). Learning to lose control: A process-based account of behavioral addiction. In *Neuroscience and Biobehavioral Reviews* (Vol. 108, pp. 771–780). Elsevier Ltd. https://doi.org/10.1016/j.neubiorev.2019.12.025

Polanía, R., Nitsche, M. A., & Ruff, C. C. (2018). Studying and modifying brain function with non-invasive brain stimulation. *Nature Neuroscience*, *21*(2), 174–187. https://doi.org/10.1038/s41593-017-0054-4

Smith, D. V., Clithero, J. A., Boltuck, S. E., & Huettel, S. A. (2014). Functional connectivity with ventromedial prefrontal cortex reflects subjective value for social rewards. *Social Cognitive and Affective Neuroscience*, *9*(12), 2017–2025. https://doi.org/10.1093/scan/nsu005

Van Holst, R. J., Van Den Brink, W., Veltman, D. J., & Goudriaan, A. E. (2010). Brain imaging studies in pathological gambling. *Current Psychiatry Reports*, *12*(5), 418–425. https://doi.org/10.1007/s11920-010-0141-7

van Holst, R. J., van den Brink, W., Veltman, D. J., & Goudriaan, A. E. (2010). Why gamblers fail to win: A review of cognitive and neuroimaging findings in pathological gambling. *Neuroscience and Biobehavioral Reviews*, *34*(1), 87–107. https://doi.org/10.1016/j.neubiorev.2009.07.007

Zheng, Y., Yi, W., Cheng, J., & Li, Q. (2020). Common and distinct electrophysiological correlates of feedback processing during risky and ambiguous decision making. *Neuropsychologia*, *146*. https://doi.org/10.1016/j.neuropsychologia.2020.107526
